# Submitral aneurysm: An unusual localization of aneurysm complicating a myocardial infarction: A case report

**DOI:** 10.1016/j.amsu.2021.103042

**Published:** 2021-11-11

**Authors:** Saîda Amaqdouf, Hammam Rasras, Anas Hbali, Soumia Boulouiz, Nabila Ismaili, Noha El Ouafi

**Affiliations:** a Department of Cardiology, Mohammed VI University Hospital of Oujda, Mohammed First University of Oujda, Morocco; b Laboratory of Epidemiology, Clinical Research and Public Health, Faculty of Medicine and Pharmacy, Mohammed the First University of Oujda, Morocco

**Keywords:** Aneurysm, Left ventricle, Submitral, Thrombus, Arrhythmia, Pericardial effusion, Case report

## Abstract

**Introduction:**

and importance: Submitral aneurysms are an uncommon heart condition that is widespread among young black Africans, congenital etiology is the most common, when SMA is found in patients of other races, other etiologies must be sought, especially ischemic.

**Case presentation:**

We present the case of a 65-year-old male patient admitted for wide complex tachycardia at a rate of 198 bpm, in whom transthoracic echocardiography revealed a submitral aneurysm and coronary angiography revealed an occlusion of the left circumflex artery.

**Clinical discussion:**

Submitral left ventricular aneurysm is a rare cardiac pathology with a variety of causes including inflammation, infection, traumatic illness, or, in rare cases, ischemic heart disease; it can be caused by a congenital defect in the posterior portion of the mitral annulus, which is more common in African population. Clinical manifestations are frequently serious and alarming, such as ventricular tachycardia, cardiogenic shock or an embolic phenomenon; however, asymptomatic cases are possible.

**Conclusion:**

What we can retain from our case is that SMA can be the cause or consequence of coronary ischemia, so we must be vigilant in patients with an atypical clinical presentation.

## Highlights

∙ Submitral aneurysms are a rare cardiac abnormality
∙ They are more common in young black Africans
∙ Congenital etiology is the most implicated but when they are found in other races, other possible etiologies must be researched.

## 1 Introduction

Submitral aneurysms of the left ventricle may be congenital or may occur after a myocardial infarction, traumatic or endocarditis. We report a case of a thrombosed submitral aneurysm following a lateral myocardial infarction that was complicated by heart arrhythmia and mitral regurgitation. The clinical conditions, aneurysm size, or other findings determine whether to follow nonsurgical strategy with close medical supervision or perform surgery which is the definitive management.

### 1.1 Patient information

A 65-year-old white male patient, a chronic smoker, with no prior medical history, was admitted to the emergency department for palpitations and epigastralgia. He stated that he had been experiencing these symptoms for the previous two weeks, but they became more severe and prolonged on the day of admission.

**Fig. 1:**
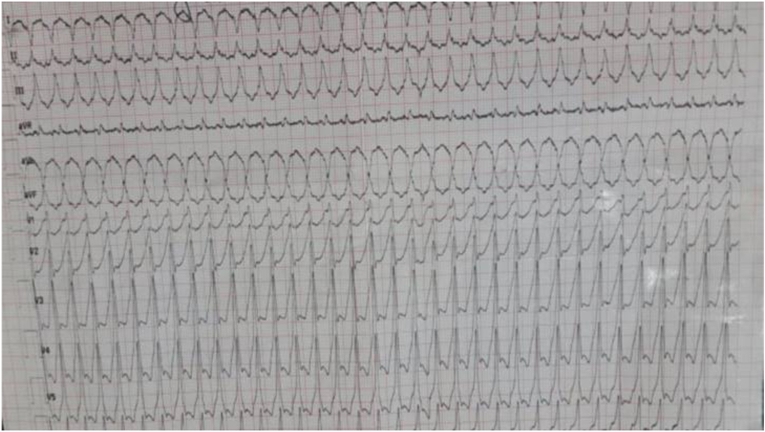
EKG showing a wide complex tachycardia at a rate of 198 bpm.

**Fig. 2:**
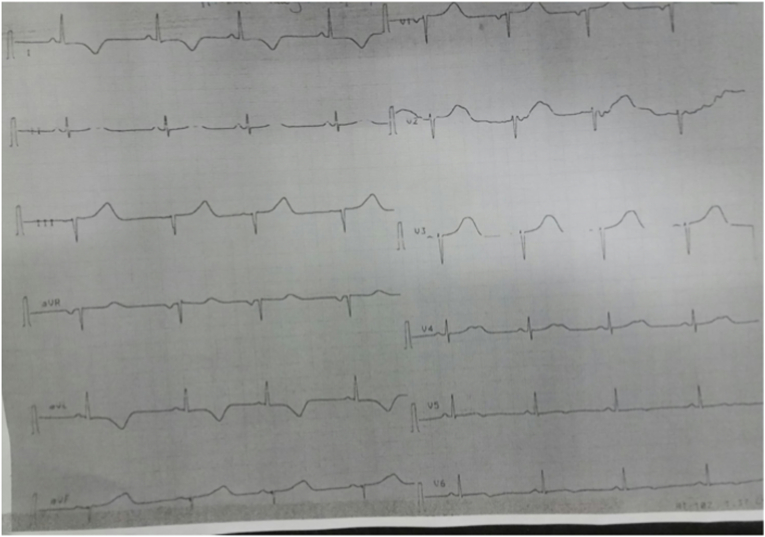
EKG after electric cardioversion.

Clinical examination showed a heart rate at 198 bpm, blood pressure was 130/60 mmHg with oxygen saturation was 95% on room air. A per-critical electrocardiogram (EKG) revealed a broad-complex tachycardia consisting with ventricular tachycardia t at a rate of 198 bpm (Fig. 1). Cardiac auscultation was difficult due to the rapid rhythm, but there were no signs of heart failure. After few minutes, the patient began to alter his state of consciousness, which justified an external electric shock at 150 J, allowing an immediate restoration of a sinus rhythm at 70 bpm. The EKG after cardioversion revealed necrotic Q waves in the inferior leads and negative T waves in the lateral leads (Fig. 2).

**Fig. 3:**
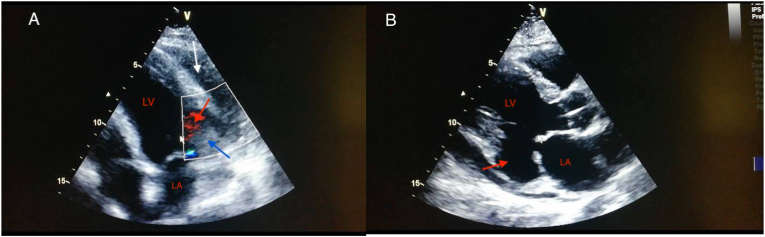
TTE; A: apical four chamber showing the aneurysm (red arrow), the thrombus (blue arrow) and the pericardial effusion (white arrow). B: parasternal long axis view demonstrating the submitral aneurysm. LV: left ventricle, LA: left atrium. (For interpretation of the references to colour in this figure legend, the reader is referred to the Web version of this article.)

**Fig. 4:**
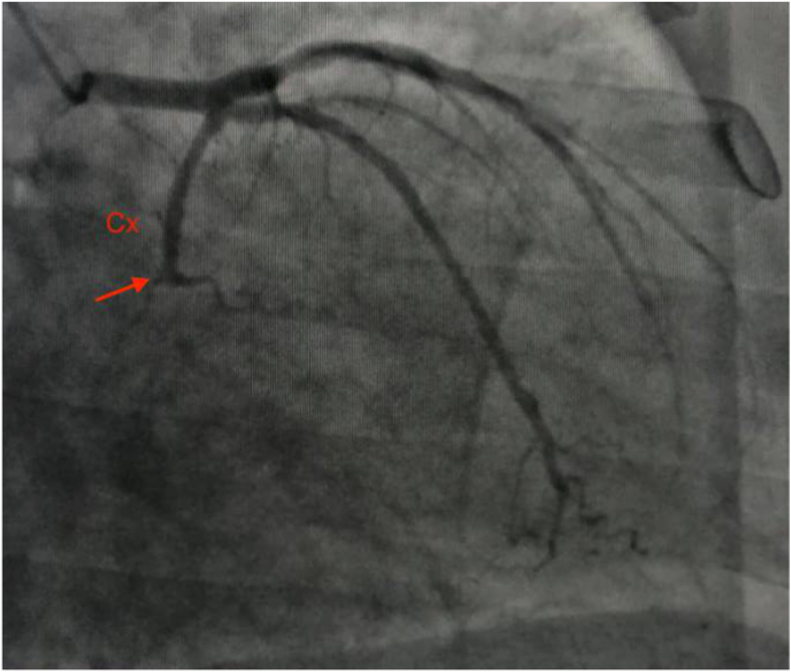
Coronary angiography: occlusion of the left circumflex artery. Cx: Left circumflex artery.

Transthoracic echocardiography showed ischemic heart disease, with a left ventricular ejection fraction of 50%, akinesia of the basal segments of the anterolateral and inferolateral walls, and minimal mitral regurgitation. Apical 4-chamber view revealed a large cavity (30 × 21 mm in diameter) adjacent to the mitral valve communicating with the left ventricle via single neck, but not with the left atrium, clogged with thrombus and moderate pericardial effusion not compromising right ventricular function (Fig. 3). Biological analysis (before electric shock) showed a significant increase in troponin and manifest inflammatory syndrome. The coronary angiography showed an atheromatous coronary network with occlusion of the left circumflex artery without intracoronary thrombi (Fig. 4), which was successfully treated with coronary stent implantation.

The patient was put on anti-ischemic medical treatment with continuous monitoring; there was no recurrence of symptoms or complications. Following a medico-surgical discussion, it was decided to re-evaluate the aneurysm's size in one month.

The patient was doing well at the one-month follow-up, the holter EKG analysis revealed only a few ventricular extra systoles, TTE showed a stationary aneurysm, and disappearance of the pericardial infusion. We decided not to operate on the patient based on these findings and instead to keep him under carful follow-up.

## 2 Discussion

According to a recent report, the prevalence of LV aneurysms was 0.1 percent and 33 percent were discovered in the submitral location [1]. A review of previous reports shows that SMAs occurs almost exclusively in African patients and is often associated with mitral regurgitation [2].

Although the exact incidence is unknown, most left ventricular aneurysms (LVAs) are caused by coronary atherosclerosis, with other common causes including congenital, post-cardiac surgical, and traumatic causes [3]. However, the etiology of submittal aneurysms is thought to be congenital rather than ischemic as only few case reports of SMAs could identify diseased coronary arteries as the cause.

The greatest diagnostic dilemma is distinguishing between true LVA and pseudo aneurysms, certain features of echocardiography favor one diagnosis over the other, and management is based on this determination. One of these characteristics is the location of an aneurysm; Pseudo aneurysms are most commonly located in the posterior and inferior left ventricular walls, whereas only 3% of true aneurysms are found in the posterior wall [4].

Du Toit et al. classified aneurysms into three types: type I (single localized neck), type II (multiple necks), and type III (involvement of the entire posterior mitral annulus) [5]. Although two-dimensional transthoracic echocardiography (TTE) is useful and sufficient for diagnosing a submitral aneurysm, in today's clinical practice, multimodality cardiac imaging such as three-dimensional (3D) echocardiography, LV contrast echocardiography, computed tomography (CT) and magnetic resonance imaging (MRI) have improved the definition of the defect, identified complications, and aided in treatment planning [6]. It was observed that cardiac MRI had an exceptional correlation with 2D and 3D TTE findings. Furthermore, cardiac MRI may reveal parietal thrombus within the aneurysm, and provide a better evaluation of ventricular function. Despite this, TTE is still the first-line diagnostic tool for this condition [7].

Coronary angiography is usually used to make a diagnosis and is the next step in treatment. When combined with echocardiography findings, coronary angiography can help distinguish true LVA from pseudo aneurysm and guide treatment [4]. A true aneurysm will reveal coronary arteries that cover the thinned ventricular wall on coronary angiography. A pseudo aneurysm, on the other hand, causes the classic draping pattern, in which the coronary arteries do not drape over the defect [3].

In our case, the diagnosis of a true aneurysm was easy because of the presence of an occlusion of the circumflex with collaterals in the aneurysmal area, the TTE was sufficient for diagnosis, and the thrombus was well visible, but cardiac MRI could be useful for eliminating a cleavage of the clogged wall by the thrombus.

While most LV aneurysms involve the anterior wall (85%), a small percentage involve the posterior wall. The atypical localization of the submitral aneurysm is a source of complications [4]. They can have a variety of clinical manifestations, such as heart failure, systemic thromboembolism, and ventricular wall rupture, myocardial ischemia due to coronary artery compression, ventricular arrhythmias, or even sudden cardiac death [8,9]. The presence of an expanding subvalvar aneurysm leads to distortion of the mitral annulus or the mitral cord, which is the most usual cause of mitral regurgitation [10]. The circumflex coronary artery generally lies in relation to the posteroinferior aspect of the aneurysm neck. Coronary artery compression can occur as a complication of submitral aneurysms, contributing to left ventricular dysfunction [11].

In our case, the patient has an occlusion rather than a circumflex compression with an atheromatous coronary network.

SMAs may serve as an arrhythmogenic substrate for re-entry circuits, which can manifest as a right bundle branch block or a left bundle branch block like morphology VT. Management strategies for VTs are aimed at preventing sudden death and recurrent life-threatening arrhythmias through antiarrhythmic drug therapy, electro anatomical mapping guided ablation, defibrillator implantation (if ablation is ineffective), or, less frequently, aneurysmectomy [5,8,12]. Surgical repair was first described in 1958 by Cooley involving a linear repair of the defect. Methods have since evolved, with patch remodeling techniques now being widely used and considered to be more efficacious by some [13]. The clinical presentation and findings of each individual patient determine the treatment modalities.The indication for surgery is determined by the size of the aneurysm, its location, the associated complications, and the risk of rupture. The clinical conditions, size of the aneurysm, or related findings determine whether to follow a nonsurgical strategy with careful follow-up or perform surgery which is the definitive management.

As our patient remained stable during his hospitalization, we decided to discharge him on medical treatment. At one month control, the TTE re-evaluation revealed a stationary aneurysm with minimal mitral regurgitation and disappearance of the pericardial infusion. Based on the findings, we decided not to operate on the patient and instead to keep him under close medical supervision.

Our case has been reported in line with THE SCARE 2020 criteria [14].

## 3 Conclusion

Submitral aneurysms are a rare cardiac abnormality; they are more common in young black Africans, whose congenital etiology is the most implicated; when they are found in other races, other possible etiologies must be researched on a case-by-case basis. Our patient had cardiovascular risk factors, and coronary angiography confirmed the ischemic origin of the aneurysm.

## Sources of funding

This research did not receive any specific grant from funding agencies in the public, commercial, or not-for-profit sectors.

## Ethical approval

Institutional review board approval is not required for deidentified single case reports or histories based on institutional policies.

## Consent

Written informed consent was obtained from the patient for publication of this case report and accompanying images. A copy of the written consent is available for review by the Editor-in-Chief of this journal on request.

## Guarantor

Saîda Amaqdouf.

## Provenance and peer review

Not commissioned, externally peer-reviewed.

## Author contribution

Saîda Amaqdouf: Study concept, Data collection, Data analysis, Writing the paper. Hamam Rasras: Data analysis, Contributor. Anas Hbali: Data analysis,Contributor. Soumia Boulouiz: Data analysis, Contributor. Nabila Ismaili: Supervision and data validation. Noha El Ouafi: Supervision and data validation.

## Registration of research studies

This is not an original research project involving human participants in an interventional or an observational study but a case report. This registration is not applicable.

## Declaration of competing interest

The authors declared no potential conflicts of interest with respect to the research, authorship, and/or publication of this article.
